# Staufen1 Protein Participates Positively in the Viral RNA Replication of Enterovirus 71

**DOI:** 10.3390/v11020142

**Published:** 2019-02-08

**Authors:** Young-Mao Chen, Bo-Ting Ou, Chao-Ying Chen, Han-Hsiang Chan, Chih-Jung Chen, Robert YL Wang

**Affiliations:** 1 Bachelor Degree Program in Marine Biotechnology, College of Life Sciences, National Taiwan Ocean University, Keelung 20224, Taiwan; ymc868@mail.ntou.edu.tw; 2 Center of Excellence for the Oceans, National Taiwan Ocean University, Keelung 20224, Taiwan; 3 Division of Microbiology and Immunology, Graduate Institute of Biomedical Sciences, College of Medicine, Chang Gung University, Tao-Yuan 33302, Taiwan; niki2011@mail.cgu.edu.tw (B.-T.O.); h318joe@gmail.com (H.-H.C.); 4 Department of Biomedical Sciences, College of Medicine, Chang Gung University, Tao-Yuan 33302 Taiwan; ying19950811@gmail.com; 5 Division of Pediatric Infectious Diseases, Department of Pediatrics, Chang Gung Memorial and Children’s Hospital, Linkou 33305 Taiwan; james.ped@gmail.com

**Keywords:** Staufen1 protein, enterovirus 71, RNA binding protein, viral translation

## Abstract

The double-stranded RNA-binding protein Staufen1 (Stau1) has multiple functions during RNA virus infection. In this study, we investigated the role of Stau1 in viral translation by using a combination of enterovirus 71 (EV-A71) infection, RNA reporter transfection, and in vitro functional and biochemical assays. We demonstrated that Stau1 specifically binds to the 5′-untranslated region of EV-A71 viral RNA. The RNA-binding domain 2-3 of Stau1 is responsible for this binding ability. Subsequently, we created a Stau1 knockout cell line using the CRISPR/Cas9 approach to further characterize the functional role of Stau1’s interaction with viral RNA in the EV-A71-infected cells. Both the viral RNA accumulation and viral protein expression were downregulated in the Stau1 knockout cells compared with the wild-type naïve cells. Moreover, dysregulation of viral RNA translation was observed in the Stau1 knockout cells using ribosome fractionation assay, and a reduced RNA stability of 5′-UTR of the EV-A71 was also identified using an RNA stability assay, which indicated that Stau1 has a role in facilitating viral translation during EV-A71 infection. In conclusion, we determined the functional relevance of Stau1 in the EV-A71 infection cycle and herein describe the mechanism of Stau1 participation in viral RNA translation through its interaction with viral RNA. Our results suggest that Stau1 is an important host factor involved in viral translation and influential early in the EV-A71 replication cycle.

## 1. Introduction

Enterovirus 71 (EV-A71) is a virus that infects humans, causes neurological diseases, and has severe effects in children [1,2]. Currently, EV-A71 is a major public health issue across the Asia-Pacific region [3]. EV-A71 is transmitted mainly through oral-fecal aerosol and droplet routes [4,5]. Humans are the only known natural host for EV-A71 infection. In general, adults and older children infected with EV-A71 are asymptomatic or exhibit only a mild form of the disease, and this form can be overcome by the human immune system. In young children, EV-A71 infection can invade the central nervous system and cause acute neurological complications, such as aseptic meningitis and rhombencephalitis, and can even lead to death [6,7,8,9]. No specific antiviral drug for the treatment of EV-A71 infection has been approved.

EV-A71 belongs to the *Enterovirus* genus and *Picornaviridae* family, which are nonenveloped viruses with a single-strand, positive-sense RNA genome that contains approximately 7500 base nucleotides [10]. Like other plus-stranded, (+)RNA viruses, EV-A71 contains positive-sense viral RNA that is similar to mRNA, and it can be immediately translated by the host’s translational machinery after entering cells [2,4,11]. Upon entering a host, the viral RNA is translated into a polyprotein. Unlike cellular cap-dependent translation, the translation of a viral protein is IRES (internal ribosomal entry site)-dependent and mediated by the IRES located in the 5′-untranslated region (UTR) of the EV-A71 RNA genome [12]. Many studies have demonstrated that the cloverleaf structure of IRES interacts with various host cellular factors, known as the IRES trans-acting factors, which can recruit the ribosome for the translation of polyproteins [13]. Several heterogeneous ribonucleoproteins (hnRNPs) have been reported to participate in the regulation of viral IRES activity, including hnRNP A1 [14], poly(rC)-binding protein 2 [15,16], polypyrimidine tract-binding protein [17], and AU-rich element binding factor 1 [18]. Among these hnRNP family proteins, hnRNP A1 may play a crucial role in facilitating EV-A71 translation. hnRNP A1 binds to the stem loop II of IRES with high affinity to promote viral RNA translation [19]. Interestingly, misshapen NCK-related kinase, a STE20 family kinase, is also involved in the regulation of hnRNP A1 translocation and IRES-dependent translation during EV-A71 infection [20]. 

Staufen is a double-stranded (dsRNA) and tubulin-binding protein. In mammalian cells, two homologues of Staufen, namely Stau1 and Stau2, have been identified as exhibiting a 51% homology with amino acid residues [21]. Stau1 contains four dsRNA-binding domains (RBDs), and dsRBD2 to dsRBD4 were reported to have the capability of binding dsRNA [22]. Stau1 was reported to bind cellular mRNA in order to form RNPs that control mRNA translation and trafficking and even regulate degraded RNA molecules [23]. Stau1 is crucially involved in the translation and degradation of cellular mRNA molecules. Generally, Stau1 enhances the efficiency of translation activity through its binding activity to the 5′-UTR of cellular mRNAs and increases the number of polysome-containing mRNA molecules. Conversely, Stau1 promotes the degradation of mRNA by binding itself to 3′-UTR of cellular mRNA targets. This process is known as Staufen-mediated mRNA decay. Stau1 degrades mRNA containing incorrect translation termination codons with a specific STAU1-binding site downstream of their normal termination sequence [24]. 

The presence of Stau1 has been reported in the infection cycles of a number of RNA viruses, including Hepatitis C virus (HCV), influenza A virus, and HIV-1. In the HCV infection cycle, Stau1 was demonstrated to be involved in the viral replication, translation, or trafficking of the HCV genome, but not in the nucleocapsid assembly [2,25]. Additionally, various studies have shown that Stau1 binds to the 3′-UTR of the HCV RNA genome as well as the negative-stranded HCV RNA intermediate to facilitate viral translation [26,27]. Stau1 has also been revealed to have an association with the HIV-1 Gag precursor protein to facilitate the processes of multimerization of the Gag protein and be bound to the HIV-1 RNA genome to enable the encapsidation of HIV-1 RNA during the assembly of viral particles [24,28]. Stau1 was reported to be a part of the Influenza A virus RNP complex and considered to facilitate the encapsidation of the viral RNA into nascent viral particles [29]. As described, we believe that Stau1 is required for RNA virus infection and may be involved in genome replication, viral protein translation, and even the assembly of viral particles.

In the present study, we demonstrated that Stau1 and Stau1 RBD2-3 binds specifically to the EV-A71 5′-UTR of the RNA genome. Through analyzing Stau1-knockout cells and viral RNA-Stau1 colocalization studies, we provide evidence that Stau1 is involved in the translation of viral RNA during the EV-A71 infection cycle. By binding to the 5′-UTR of the EV-A71 RNA genome, Stau1 recruits more viral RNAs in the ribosome-containing complex as performed by the polysome fraction and prolongs the viral RNA’s stability, thereby facilitating the process of viral translation. Overall, these findings indicate that Stau1 is an important host factor participating in translation and replication steps during the EV-A71 infection cycle. The results also provide a new route of knocking down the essential host factor as an antiviral strategy.

## 2. Materials and Methods

### 2.1. Cells and Virus

Human rhabdomyosarcoma (RD) cells were cultured with Dulbecco’s modified Eagle medium (DMEM) (Gibco-BRL, Carlsbad, CA, USA) which was supplemented with 10% FBS (Gibco-BRL), 100 units penicillin, 50 μg/mL streptomycin (Gibco-BRL), and 24 mM sodium bicarbonate (Sigma, St. Louis, MO, USA), and maintained at 37 °C in an atmosphere of 5% CO_2_. EV-A71 ((TW/2231/98) was isolated in the EV-A71 outbreak and supplied by the Clinical Virology Laboratory of Chang Gung Memorial Hospital, Taiwan) was amplified in RD cells and the titers were measured by plaque assay. For virus infection, cells were washed with PBS, and incubated in serum free medium 1 h prior to infection. Cells were infected by incubation with EV-A71 for 1 h at 37 °C, followed by removing the virus and washing with PBS, and cultured in the complete medium as described above. 

### 2.2. Plasmids Construction

The pEV-A71 5′-UTR and 3′-UTR were constructed as follows: The 5′-UTR of EV-A71 was amplified by PCR from the EV-A71 full-length infectious cDNA clone using #W416 5′-(TAATACGACTCACTATAGGGAGATTAAAACAGCCTGTGGGT); #W417 5′-(GTTTGATTGTGTTGAGGGTC) and the primers for 3′-UTR using #W418 5′-(TAATACGACTCACTATAGGGAGAATTTACAGTTTGTAACTG); #W419 5′-(GCTATTCTGGTTATAACAAA), which contained the T7 promoter. The Blunt-end PCR products generated by proofreading DNA polymerases could be directly ligated with the pZBack/blunt linearized vector (BioTools Co., Ltd, Taipei, Taiwan). The GAPDH 5′-UTR was constructed as follows: The 5-terminus untranslated region (188 bp) of Glyceraldehyde-3-phophate dehydrogenase (GAPDH, NCBI reference: NM_002046.5) was amplified by RT-PCR from total RNA isolated from HeLa cells using #W487 5′-(TAATACGACTCACTATAGGGGCCTCAAGACCTTGGGCTG) and #488 5′-(GGTGTCTGAGCGATGTGGC), followed by cloning in the pZBack/blunt lineared vector as described above. The Stau1 full length and different truncated PCR products were then treated with *Sal*I, *Not*I, following by ligation into the pGEX vector, which was designed to produce GST-binding protein (GST) fusions, (GE Healthcare Bio-Sciences Co., Ltd, Piscataway, NJ, USA) that were previously digested with the restriction enzymes as shown above.

### 2.3. RNA-Protein Immunoprecipitation

For synthesis of biotin-labeled RNA transcript, the EV-A71 genomic regions, 5′-UTR and 3′-UTR were constructed in pZback/blunt linearized vector (BioTools Co., Ltd). The blunt-end PCR products generated by proofreading DNA polymerases can be directly ligated with the pZBack/blunt vector, followed by the in vitro transcription. RNA transcripts were synthesized using the MEGAscript T7 kit (Thermo Fisher Scientific Co., Ltd, Waltham, MA, USA) according to the manufacturer’s protocol. In brief, the biotinylated RNA was produced in an 20 μL MEGAscript transcription reaction with 20 mM biotinylated UTP. The synthesized RNAs were then purified and analyzed on 1% agarose gel. To carry out the co-immunoprecipitation, 8 μg of biotinylated RNAs were added into Streptavidin Sepharose High Performance beads (GE Healthcare), following by adding the recombinant MBP-Stau2 protein and incubation for 4 h. This reaction received the UV cross-link in the UV CROSSLINKER (Spectroline, Westbury, NY, USA) to enhance the binding of RNA and recombinant protein. The bound eluates were then analyzed by western blotting. 

### 2.4. Establishment of Stau1-Knockout RD Cells by CRISPR/Cas9 System

Human rhabdomyosarcoma (RD) cells were cultured in Dulbecco’s modified Eagle medium (DMEM) supplemented with 10% fetal bovine serum. Three expression plasmids for Cas9 gene, single guide RNA (sgRNA) for human Stau1 and reporter (CMV-RFP-sgRNA target-hygromycin-EGFP) were designed and purchased from ToolGen, Inc. Stau1 sgRNA target sequence: TTTGTGACCAAGGTTTCGGTTGG, underlined are PAM sequence. Cells were co-transfected with Cas9, sgRNA and reporter plasmids using Lipofectamine LTX & PLUS™ Reagent (Thermo Fisher Scientific) at a weight ratio of 2:2:1 (Cas9: sgRNA: reporter). Two days after transfection, transfection efficiency was determined by RFP expression, dRGEN activity was determined by EGFP expression using fluorescence microscopy and validated by T7E1 assay. Stau1-knockout cells were selected with 2 mg/mL of hygromycin B at 37 °C for three days and collected. The limiting dilution of cells cultivation was performed in order to establish STAU1 knockout clones. In brief, seeding 1 cell per well in the 96-well plates and harvesting the cells after two to four weeks, followed by expanding individual cell colonies. The cells were then harvested for extraction of genomic DNA and subjected to fluorescence PCR (F-PCR) for genotyping analysis and total cell lysates were subjected for validation of protein expression level by western blotting using anti-Stau1 specific antibody.

### 2.5. Polysome Fractionation Assay

The collection of the EV-A71-infected normal and Stau1-knockout cells was carried out with cold PBS containing 100 μg/mL cycloheximide and centrifugation at 1000× *g* for 5 min at 4 °C. The cell pellet was re-suspended in 1 mL of RSB150 (10 mM Tris-HCl (pH7.4), 3 mM MgCl_2_, 150 mM NaCl, 100 μg/mL cycloheximide), containing 35 μg/mL digitonin, 20 U/mL RNase inhibitor and protease inhibitors. The cell lysates were then incubated on ice for 5 min, followed by using the 26 gauge needles to break out the cells and isolate the pellet nuclei and debris at 3000× *g* for 1 min at 4 °C. The supernatant lysates were subjected to the sucrose gradient experiment, which was carried out 15 to 40% sucrose separating 12 fractionations by ultra-centrifugation for 3 h (40,000 rpm in the Corning^®^ CentriStar™ Centrifuge Tubes, Beckman SW41 Ti rotor, Beckman Coulter, Indianapolis, IN, USA). RNA isolated from these 12 fractions were then subjected to RT-qPCR for analysis of the RNA quantitation in each fraction.

### 2.6. Plaque Assay

SF268 cells were seeded in 6-well plates at 4 × 10^5^ cells per well, followed by an overnight incubation in RPMI 1640 medium containing 5% FBS to form a monolayer. The supernatants of culture medium samples from EV-A71-infected cells and 10-fold serial dilutions of the sucrose gradient fractions were prepared in serum-free DMEM medium before infection. Monolayer SF268 cells were incubated with serum-free DMEM medium for 1 h before adding 0.5 mL of the 10-fold dilutions and 0.5 mL of the serum-free DMEM medium. The SF268 cells were incubated for an additional hour to allow infection to occur. The cells were washed with the serum free medium, and 2 mL 0.3% agarose (Invitrogen, Waltham, MA, USA) in serum free medium was added to each well. The plates were incubated at room temperature for 30 min to solidify the agarose, and cells were cultured at 37 °C for 4 day. The cells were fixed with 2 mL of 10% formaldehyde, and incubated for 30 min at room temperature before the agarose overlays were removed. The cells were stained with a solution of 0.5% crystal violet, 1.85% formalin, 50% ethanol, and 0.85% NaCl (Sigma) for 2 min, and washed with deionized water. Virus titers were calculated as the number of plaques X (1 mL/0.5 mL) X the serial dilution factor, and were expressed in PFU/mL.

### 2.7. IRES Activity Assay

The EV-A71 IRES domain II-IV region was subcloned into pGL4.17 vector followed by co-transfection of pGL4.17-IRES and pcDNA3.1-Rellina in the RD cells. At 24 h post-transfection, the cells were harvested by 1× passive lysis buffer (supplied by the manufacturer) for the dual-luciferase reporter assay according to the manufacturer’s protocol (Dual-Luciferase^®^ Reporter Assay System (Promega, Madison, WI, USA)). In brief, 100 μL of the firefly luciferase reagent was added to the cell lysates, with a 10-s equilibration time and measurement of luminescence. Then, the same tube was added to the *Renilla* luciferase reagent (100 μL) and firefly quenchingand measurement of luminescence with a 10-s integration time. The data were represented as the ratio of firefly to *Renilla* luciferase activity (Fluc/Rluc). 

### 2.8. Western Blotting and Antisera

The procedures for protein sample collection and separation have been described elsewhere [30]. In brief, the equal amount of total proteins were heated for 3 to 5 min, followed by separation in the 12% SDS-PAGE under the reduced conditions. After the separation, the gel was then transferred to a polyvinylidene difluoride (PVDF) membrane (Bio-Rad Laboratories, Hercules, CA, USA) for the primary and secondary antibodies incubation. The primary antibodies used were listed as the following: Rabbit anti-GST (GeneTex Inc, Irvine, CA, USA, 1:1000), anti-Flag (Sigma, 1:3000 dilution) and anti-EV-A71 3D (1:5000 dilution); for detection of Stau1 (GeneTex, 1:2000) and β-actin (Sigma, 1:10,000). The primary and secondary antibodies were diluted in the Super antibody mate solution (MDBio Co., Ltd, Taipei, Taiwan) followed by incubation with the membranes. 

### 2.9. Immunofluorescent Staining

Immunofluorescent staining of Stau1, dsRNA and viral proteins has been described elsewhere [31]. The primary antibodies used in our study were anti-Stau (Enzo Life Science, Farmingdale, NY, USA), and anti-EV-A71 (Millipore, Billerica, MA, USA). Rhodamine or fluorescein isothiocyanate (FITC) conjugated secondary antibodies were used. Images were acquired using a Zeiss confocal microscope (ZEISS medical technology Co., Ltd, Athens, Greece), and processed with Adobe Photoshop software (Adobe Photoshop CS6, Adobe, San Jose, CA, USA).

### 2.10. RNA Preparation, Reverse Transcription, and Quantitative Real-Time PCR

Total RNA was isolated from the TRIzol (Invitrogen) reagent according to the manufacturer’s protocol. Random primers, reverse transcriptase (ABI), and 3 μg of total RNA were used to produce the cDNA. Quantifications of the Stau and the EV-A71 cDNAs were performed by quantitative real time PCR (qPCR) using the Stau forward and reverse primers, CAGAATGAAGGAAAACCAGAAGC and GCACACAATACTCATCAATGGG, respectively, and the EV-A71 forward and reverse primers, TCAATTCCCGTTTCTCATCCA and GAGGGAGCGCACGTGATCT, respectively. The qPCR analyses were performed in duplicate using SYBR green master mix (KAPA) in an ABI 7500 qPCR system.

## 3. Results

### 3.1. Human Stau1 Protein is Associated with Viral RNA in the EV-A71-Infected Cells

**Figure 1:**
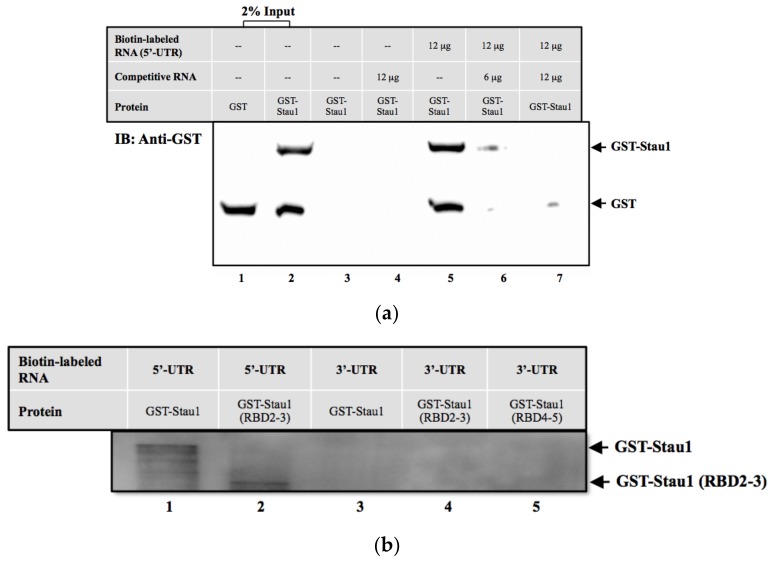
Stau1 binds to 5′ untranslated region of EV-A71 RNA. (**A**) Stau1 binds to EV-A71 viral RNA in an in vitro RNA pulldown assay. The recombinant GST-tagged Stau1 protein was expressed and purified from *Escherichia Coli*. The biotin-labeled EV-A71 viral RNA was used for the RNA pulldown analysis. Lanes 1 and 2: Input of the recombinant GST or GST-Stau1 protein; Lane 3: GST-tagged Stau1 protein only; Lane 4: GST-tagged Stau1 protein interacted with non-biotin-labeled RNA (competitive RNA); Lane 5–7: GST-tagged Stau1 protein interacted with biotin-labeled RNA without or with competitive RNAs. (**B**) Stau1 binds to 5′-UTR but not 3′-UTR of EV-A71 RNA genome. GSI-tagged Stau1 protein was expressed for the in vitro RNA pulldown assay as described above. The GST-Stau1 (RBD2-3) is a truncated protein containing RNA-binding domain 2 and 3 as described in Section 3.2.

**Figure 2:**
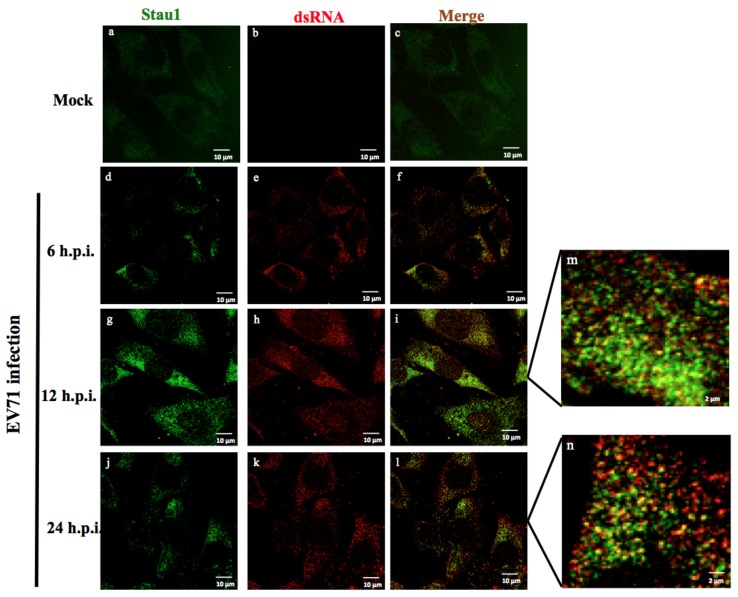
Colocalization of Stau1 with viral RNA in EV-A71-infected cells. RD cells were infected without or with EV-A71 at an MOI of 1 for 6, 12, and 24 h, followed by fixation and coimmunostaining with anti-Stau1 (green) and anti-dsRNA (red) antibodies. Bound primary antibodies were detected using secondary antibodies conjugated to Texas Red (for dsRNA) or fluorescein isothiocyanate (for Stau1). The images represent three independent experiments that used three independent infections. The merged images of anti-Stau1 (green) and anti-dsRNA (red) are shown in panels c, f, i and l, respectively. Panels m and n are enlarged images of panels i and l, respectively. Bar = 10 µm.

It has been reported that some host RNA-binding proteins (such as: Stau1, hnRNPU, DDX3, and La proteins) interact with the viral RNA genome during the RNA viruses infection cycle [14,19,32]. Among these proteins, we selected Stau1, which contained four featured RNA-binding motifs, for further characterization. First, we conducted RNA protein immunoprecipitation followed by Western blotting to verify that Stau1 was associated with the 5′-UTR of viral RNA (Figure 1a). We demonstrated that recombinant Stau1 protein binding was preferred to 5′-UTR of viral RNA, but was not observed in the 3′-UTR RNA transcript (Figure 1b). In addition, we revealed the colocalization of Stau1 with viral dsRNA using dual immunofluorescent staining (Figure 2). Because viral dsRNA is an intermediated product during viral RNA replication, this result suggested that Stau1 protein was also involved in the viral replication complex in EV-A71-infected cells.

### 3.2. Stau1 Protein Interacts with Viral RNA through Its RNA-Binding Domain

**Figure 3:**
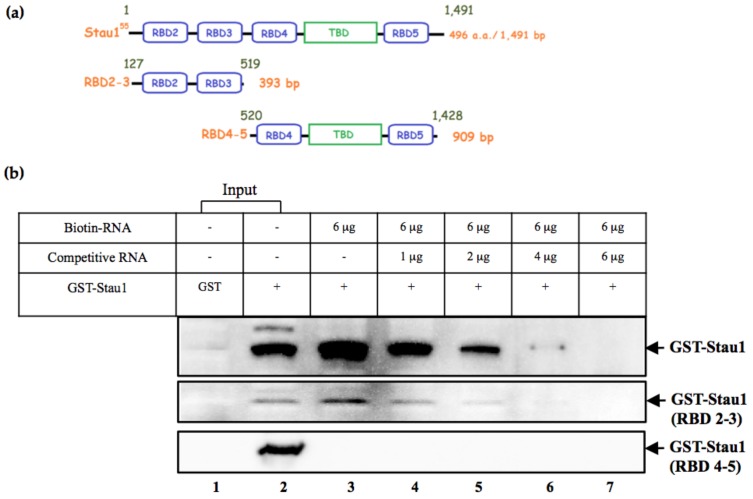
Stau1 interacts with EV-A71 viral RNA through its RBD2-3. (**A**) Domain structure of Stau1. The regions RBD2-3 and RBD4-5 are indicated with superscript numbers (nt). (**B**) Pulldown experiments using streptavidin beads directed against biotin-labeled RNA. Binding of an RNA-specific protein was determined using an anti-GST antibody (Lane 3). The non-biotin-labeled RNA was used as the competitive RNA (Lane 4–7). The input of the GST-Stau1 recombinant protein is shown as the control (Lane 2). The “+” indicates the addition of recombinant GST-Stau1 protein; the “−“ indicates no biotin-RNA/competitive RNA.

There are four RNA-binding domains in Stau1. To determine which domains are responsible for EV-A71 viral RNA binding ability, we constructed two fragments of Stau1 in vitro: RNA-binding domain 2 and 3 (RBD2-3) and RNA-binding domain 4 and 5 (RBD4-5) of recombinant proteins (Figure 3A). The expression of the two Stau1 truncated proteins was then examined for RNA-binding ability through the in vitro synthesized RNA transcripts and using an RNA pulldown assay. As illustrated in Figure 3B, the full length of recombinant Stau1 was bound to biotin-labeled viral RNA, and this binding was competed by the addition of non-biotin-labeled RNA, revealing the specific binding between Stau1 and viral RNA. Next, only the RBD2-3 Stau1 protein was bound to biotin-labeled RNA and was completed by non-biotin-labeled RNA. This result suggested that Stau1 interacts with EV-A71 viral RNA through its RNA-binding domain 2-3 amino acid regions (Figure 3B). 

### 3.3. Reduction of EV-A71 Viral RNA Replication in the Stau1-Knockout Cells

**Figure 4:**
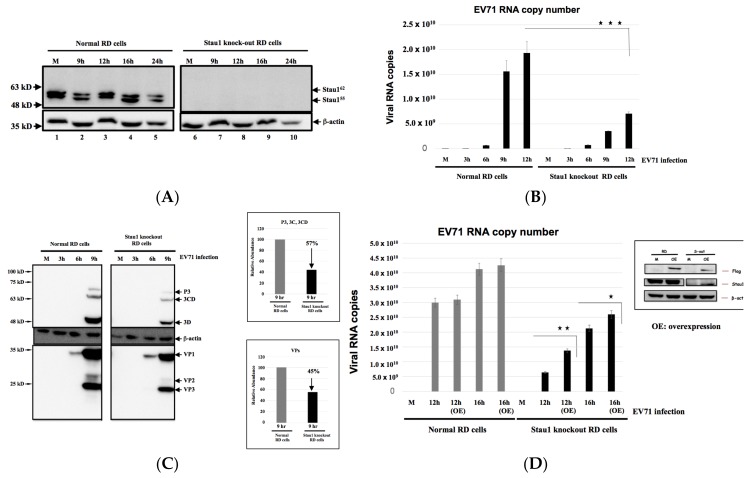
Reduction of EV-A71 viral RNA accumulation and viral protein production in the Stau1-knockout cells. (**A**) The Stau1 knockout cell line was generated using the CRISPR/Cas9 system as indicated in the Materials and Methods section. No expression of Stau1 was confirmed in the cells upon EV-A71 infection. (**B**) Reduction in EV-A71 viral RNA was determined using RT-qPCR analysis in the EV-A71-infected normal cells and Stau1 knockout cells. (**C**) Decreased production of EV-A71 viral proteins in the EV-A71-infected normal cells and Stau1 knockout cells. The right panels are the relative densities of protein bands measured using ImageJ software. (**D**) Ectopic expression of Stau1 restored the EV-A71 viral RNA accumulation in the Stau1 knockout cells. Overexpression of Flag-tagged Stau1 protein was detected during the Western blotting using anti-Flag- and anti-Stau1-specific antibodies in normal cells and Stau1 knockout cells (right panel). Star sign * indicates significant difference in comparisons * *p* < 0.05; ** *p* < 0.01; *** *p* < 0.001. OE: Overexpression; M: Transfection with plasmid only.

To reveal the functional relevance of Stau1 in the EV-A71 infection cycle, we created Stau1 knockout cells. RD cells were transfected with Cas9-encoding plasmid followed by cotransfection of the in vitro transcribed sgRNA into the cells. The cells were then harvested at various time points post-transfection for further sequence analyses. As indicated in Figure 4A, we successfully created the Stau1 knockout cells, as there was no stau1 protein expression. EV-A71 infection was then challenged in the Stau1 knockout and normal RD cells. Interestingly, we observed a weaker viral-induced cytopathogenic effect (CPE) in the Stau1 knockout cells compared with the wild-type cells (data not shown). A decreased virus growth was observed in the Stau1 knockout cells compared with normal EV-A71-infected RD cells (Figure 4B). Additionally, the expression levels of the viral structural proteins VP1, VP2, and VP3 as well as the nonstructural proteins 3D/3CD were only 57% and 45% in comparison with the normal cells (Figure 4C), implying that Stau1 is involved in the EV-A71 infection cycle.

To further investigate the functional role of Stau1 in the viral RNA replication step, we then ectopically expressed Stau1 protein in the Stau1 knockout RD cells. The overexpression of Stau1 in both normal and Stau1 knockout RD cells was validated through Western blotting using anti-Flag- and anti-Stau1-specific antibodies, respectively (as shown in the right-hand small panel in Figure 4D). As expected, significantly increased viral RNA accumulation was detected in the Stau1-overexpressed and Stau1-knockout cells at 12 h and 16 h postinfection (Figure 4D), indicating that Stau1 protein is participating in the viral RNA replication step.

### 3.4. Stau1 Enhances IRES Activity in Vitro 

**Figure 5:**
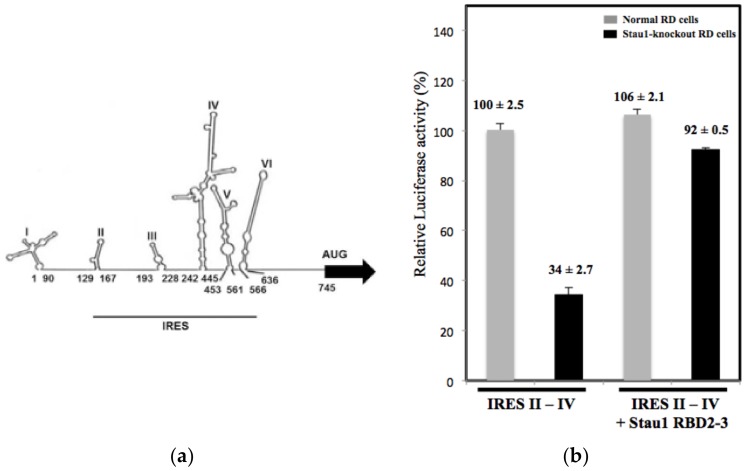
Stau1 positively regulates the EV-A71 IRES activity. (**A**) EV-A71 viral IRES genome structure. (**B**) The effect of Stau1 on the IRES activity was evaluated with IRES domain II–IV. The IRES domain II–IV was subcloned into pGL4.17 (containing Firefly luciferase gene) plasmid followed by cotransfection with pCDNA3.1-CMV (containing Renilla luciferase gene) in normal cells and Stau1 knockout cells. A dual luciferase reporter assay was performed to measure the IRES activity. Overexpression of recombinant Stau1 RBD2-3 protein was used for the restored experimental assay.

As we revealed that Stau1 protein interacted with the IRES region in the 5′-UTR of viral RNA, we hypothesized that Stau1 may participate in EV-A71 viral protein synthesis. To address this, we first constructed an IRES activity reporter plasmid (pGL4.17-IRES) to evaluate IRES activity in the RD cells (Figure 5A). 48 hours after the transfection of the IRES reporter plasmid, cell lysates were harvested and used to calculate IRES activity. As presented in Figure 5B, we detected lower IRES activity (34% lower) in the Stau1 knockout cells compared with the normal RD cells. This result indicated that Stau1 positively regulates IRES-mediated viral translation. To further confirm the positive role of Stau1 in EV-A71 IRES activity, the ectopic expression of Stau1-RBD2-3 protein was conducted in the Stau1 knockout cells to confirm the functional role of Stau1 in viral translation. The expressed Stau1 could restore the IRES activity up to three fold (from 34% to 92%, see Figure 5B), implying that Stau1 can enhance the EV-A71 viral IRES activity in RD cells, and that the RNA-binding domain 2-3 (RBD2-3) of Stau1 is responsible for this function. 

### 3.5. Stau1 Increases Viral Translation Efficiency

**Figure 6:**
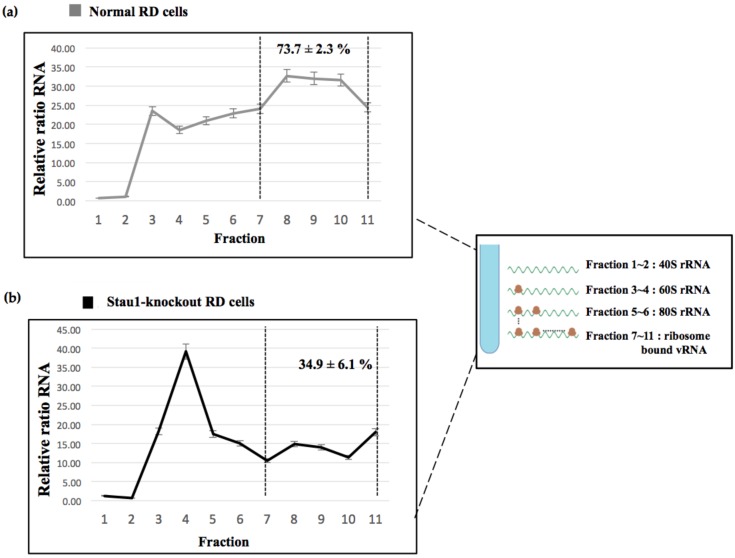
Investigation of polysome profile in the Stau1 knockout cells. The normal and Stau1 knockout RD cells were infected with EV17 at an MOI of 1. At 24 h postinfection, the cell lysates were harvested and subjected to sucrose density fractionation to obtain the polysome profile. Each fraction was harvested for RNA isolation and quantitation. (**a**) EV-A71-infected normal RD cells. (**b**) EV-A71-infected Stau1 knockout RD cells. The representative of different ribosomal RNAs and ribosome-bound mRNA (between two vertical dot lines) is shown in the enlarged panel (The wave line is referred to the different fractions).

Because Stau1 interacts with the 5′-UTR of EV-A71 viral RNA and facilitates IRES activity, we investigated whether Stau1 participates in the viral translation process. A polysome fractionation assay was employed to calculate the translational efficiency in vivo. The normal and Stau1-knockout RD cells were infected with EV-A71 at a multiplicity of infection (MOI) of 1. At 24 h postinfection, the cell lysates were harvested and subjected to sucrose density gradient centrifugation. Each fraction was collected for RNA isolation. We discovered that 74% of total viral RNA (as shown in the Figure 6 Ribosome-bound vRNA) was detected in the normal RD cells (Figure 6a). By contrast, only 35% of total viral RNA was detected in the Stau1 knockout cells (Figure 6b). These results indicated that Stau1 facilitates viral translation through the enhancement of viral IRES activity as well as increasing the viral translation efficiency. 

### 3.6. Stau1 Protein Increases Stability of the 5′-UTR of EV-A71

**Figure 7:**
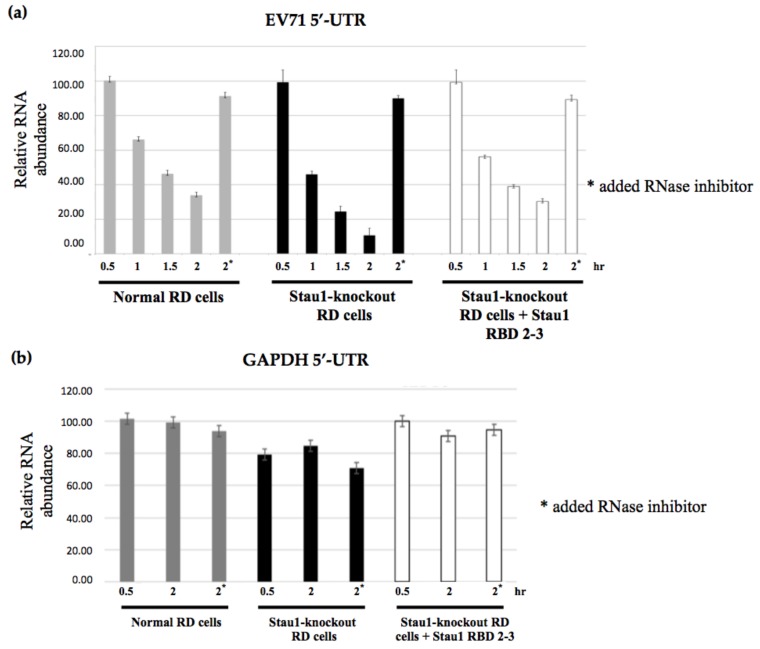
Stau1 improves the stability of 5′-UTR ofEV-A71 viral RNA. The 5′-UTR of EV-A71 RNA was transfected in the normal, Stau1 knockout, and recombinant Stau1-overexpressed RD cells for the viral RNA stability assay. The transfectant cell lysates were then harvested at 30 min posttransfection for the RNA stability assay. RNase inhibitor was added for the negative control as shown in * (**a**). The 5′-UTR of GAPDH RNA was used as the experimental control (**b**).

In general, mRNA stability is an important factor in the translational process. From the presented results, we know that Stau1 bound to the 5′-UTR of EV-A71 viral RNA, which enhanced IRES activity as well as increased the viral translational efficiency as determined by the polysome profile. Next, we hypothesized that Stau1 protein also increased viral RNA stability when bound to the EV-A71 5′-UTR of the RNA. To confirm this hypothesis, we employed RNA stability assay through transfection of the EV-A71 5′-UTR RNA transcript in both normal and Stau1 knockout cells. The cells were then harvested at various posttransfection time points and used for the quantitation of viral RNA using reverse transcriptase-quantitative PCR (RT-qPCR). The lower level of 5′-UTR of viral RNA was measured at 2 h after EV-A71 5′-UTR RNA transfection infection in the Stau1-knockout cells compared with the normal cells (Figure 7A). The ectopic expression of Stau1 RBD2-3 in the Stau1-knockout cells restored more viral RNA at the same time point (Figure 7A). To assess whether the effect of Stau1 on 5′-UTR RNA was specific, these experiments were repeated using a transfection of the 5′-UTR of the GAPDH RNA transcript (186 bp). The data indicated that there was no significant reduction of the GAPDH 5′-UTR among normal, Stau1-knockout, and Stau1-expressed RD cells (Figure 7B), which suggested that Stau1 also stabilizes the EV-A71 5′-UTR through its interaction. 

## 4. Discussion

Because the positive-sense RNA of EV-A71 is used for protein translation, it is necessary for such RNA to bind to the host ribosome for translating viral proteins [33,34] in the early stage of viral infection. Stau1 was previously identified as a double-stranded RNA-binding protein, and we believe that Stau1 influences the life cycle of EV-A71 through binding to an IRES structure, which is unique to the 5′-UTR of viral RNA (Figure 1B). In this study, we demonstrated that viral protein production was considerably reduced in Stau1-knockout cells. More importantly, the negative sense of viral RNA, an intermediate RNA template for viral RNA replication, was also decreased. These results indicated that Stau1 may facilitate the translation and replication of the EV-A71 genome in the EV-A71 life cycle. 

Stau1 is an RNA-binding protein that is present in conserved domains from the simplest to the most complex eukaryotes. Stau1 in *Drosophila* was the first to be described [32]; it influenced the localization of various RNAs by direct binding to the targeted RNAs or was involved as a component of RNP complexes [35,36]. Stau1 has been reported to contain four double-strand RNA-binding domains (RBD2 to RBD5, see Figure 3A) because RBD1 is present only in the Stau2 isoform. We identified RBD2-3 as the viral-RNA-interacting region (Figure 3B). These domains are likely to be involved in 5′-UTR viral-RNA-binding and presumably also interact with cellular proteins involved in viral translation. By contrast, the RBD4-5 of Stau1 did not interact with viral RNA or viral 3D protein (data not shown). Some studies have reported that Stau1, with its RNA complexes, can interact with the cytoskeleton through the tubulin-binding domain (TBD), which is located at its C-terminus, to be transported to their destinations, such as the endoplasmic reticulum or free polysomes [21]. In our study, we did not observe interaction between RBD4-5, which contains the TBD, and viral RNA, or the transportation of Stau1-RNA complexes within the cytoplasm, indicating that Stau1 may not regulate the trafficking of viral RNA.

Stau1 boosted the IRES-mediated viral translation of EV-A71 viral RNA in our dual luciferase reporter assay and polysome profile results. Although the particular step enhanced by Stau1 during the viral translational process remains to be determined, this effect was dependent on the presence of the RBD2-3 domain of Stau1. The 5′-UTR region of EV-A71 viral RNA that lacked Stau1 was significantly unstable, as determined by in vitro RNA stability assay, and the overexpressed RBD2-3 Stau1 protein could restore RNA stability (Figure 7), indicating that the association of Stau1 with the 5′-UTR region of viral RNA is capable of prolonging the stability of viral RNA as a template for viral translation. 

## 5. Conclusions

These results suggest that human RNA binding protein Stau1 is participating in EV-A71 replication and the regulation of viral RNA stability, as well as the production of viral protein machinery during the viral infection life cycle. Upon viral infection, in the early stage, Stau1 facilitates viral translation through binding with viral RNA. Additionally, the reduction of EV-A71-progeny viral RNA was detected in the Stau1 knockout cells, implying that Stau1 could be a positive regulator of viral infection.
